# Iron Deprivation Modulates the Exoproteome in *Paracoccidioides brasiliensis*

**DOI:** 10.3389/fcimb.2022.903070

**Published:** 2022-06-03

**Authors:** Aparecido Ferreira de Souza, Laurine Lacerda Pigosso, Lana O’Hara Souza Silva, Italo Dany Cavalcante Galo, Juliano Domiraci Paccez, Kleber Santiago Freitas e Silva, Milton Adriano Pelli de Oliveira, Maristela Pereira, Célia Maria de Almeida Soares

**Affiliations:** ^1^Laboratório de Biologia Molecular, Instituto de Ciências Biológicas, ICB II, Campus II, Universidade Federal de Goiás, Goiânia, Brazil; ^2^Instituto de Patologia Tropical e Saúde Pública, Universidade Federal de Goiás, Goiânia, Brazil

**Keywords:** secretome, cytochrome b5 (CYB5), microbial adaptation, Fe, nutritional immunity

## Abstract

Fungi of the *Paracoccidioides* genus are the etiological agents of the systemic mycosis paracoccidioidomycosis and, when in the host, they find a challenging environment that is scarce in nutrients and micronutrients, such as Fe, which is indispensable for the survival of the pathogen. Previous studies have shown that fungi of this genus, in response to Fe deprivation, are able to synthesize and capture siderophores (Fe^3+^ chelators), use Fe-containing host proteins as a source of the metal, and use a non-canonical reductive pathway for Fe^3+^ assimilation. Despite all of these findings, there are still gaps that need to be filled in the pathogen response to metal deprivation. To contribute to the knowledge related to this subject, we obtained the exoproteome of *Paracoccidioides brasiliensis* (*Pb*18) undergoing Fe deprivation and by nanoUPLC-MS^E^. One hundred forty-one proteins were identified, and out of these, 64 proteins were predicted to be secreted. We also identified the regulation of several virulence factors. Among the results, we highlight Cyb5 as a secreted molecule of *Paracoccidioides* in the exoproteome obtained during Fe deprivation. Cyb5 is described as necessary for the Fe deprivation response of *Saccharomyces cerevisiae* and *Aspergillus fumigatus.* Experimental data and molecular modeling indicated that Cyb5 can bind to Fe ions *in vitro*, suggesting that it can be relevant in the arsenal of molecules related to iron homeostasis in *P. brasiliensis*.

## Introduction

Sophisticated arsenals, elaborate tactics, and constant strategic changes: what could be the description of a war scenario also characterizes the host–pathogen interaction event (Asehnoune et al., 2016; Gonzalez and Hernandez, 2016). Among the myriad of aspects studied in the pathogen–host interaction, nutritional immunity occupies a prominent place because it consists of the innate ability of hosts to control the bioavailability of essential nutrients and micronutrients, affecting the survival of pathogens (Wang and Cheraryil, 2009; Soares and Weiss, 2015; Núñez et al., 2018).

Pathogen survivability relies on the competition between pathogen and host for micronutrients such as iron (Fe), triggered by nutritional immunity. Biologically, the micronutrient Fe is predominantly found as ferrous (Fe^2+^) and ferric ions (Fe^3+^). The flexibility between these oxidation states allows the metal to be used in several vital cellular processes such as energy metabolism, gene expression, and protein stability (Nairz et al., 2010; Dlouhy and Outten, 2013). However, it should be noted that the same flexibility of Fe oxidation states also gives a toxic potential; therefore, strict control of metal metabolism is indispensable. To this end, both pathogens and hosts employ mechanisms to maintain adequate amounts of Fe to meet their metabolic demands and counteract their toxicity, which characterizes Fe homeostasis (Tandara and Salamunic, 2012).

An increasing number of works in the literature have gradually contributed to the understanding of mechanisms that pathogenic bacteria and fungi employ for the uptake of Fe in the host (Schaible and Kaufmann, 2004; Wang and Cheraryil, 2009; Caza and Kronstad, 2013; Choi et al., 2015). Regarding pathogenic fungi, the mechanisms they use to obtain iron in the context of infection can be summarized in the following: 1) absorption of siderophores or heme/hemoglobin mediated by receptors, which characterizes non-reductive assimilation pathways of Fe, in addition to 2) a reductive Fe assimilation pathway allowed by enzymatic complexes that promote oxidation of the metal and its uptake (Bailão et al., 2012; Caza and Kronstad, 2013; Bairwa et al., 2017; Roy and Kornitzer, 2019). Some fungi employ more specific strategies, such as *Histoplasma capsulatum*, which secretes gamma-glutamyl transpeptidase (GGT) that acts in capturing Fe by generating a dipeptide with a high reducing power by cleaving glutathione (Zarnowski and Woods, 2005; Zarnowski et al., 2008). It is also known that cytochrome b5 (Cyb5) regulates the Fe metabolism of some fungi, such as *Saccharomyces cerevisiae* (Dap1) and *Aspergillus fumigatus*, in a process linked to ergosterol biosynthesis (Craven et al., 2007; Misslinger et al., 2017).

Fungi of the *Paracoccidioides* genus are the etiologic agents of paracoccidioidomycosis (PCM), an endemic systemic mycosis in Latin America whose accurate diagnosis and short-term therapeutic approach are still challenges (Shikanai-Yasuda et al., 2017). Previous studies have shown that the response of *Paracoccidioides* to Fe depletion is an important virulence attribute for the pathogen (Silva et al., 2020). Once under metal depletion, *Paracoccidioides* spp. undergoes intense adaptation of its metabolism (Parente et al., 2011). The mechanisms that the fungus uses to obtain Fe when in the host have been targets of study. *Paracoccidioides* spp. is able to synthesize and use siderophores, capturing them *via* receptors. Those fungi present orthologous genes encoding the enzymes necessary for the biosynthesis of hydroxamates, and plasma membrane proteins related to the transport of these molecules, all induced in iron deprivation (Silva-Bailão et al., 2014). *Paracoccidioides* spp. is able to use siderophores as an iron source, increasing the fungus ability to survive inside macrophages, an iron-poor environment (Silva-Bailão et al., 2014). The addition of the xenosiderophore ferrioxamine B (FOB) to *Paracoccidioides brasiliensis* culture medium results in repression of the SidA products, the first enzyme of the siderophore biosynthesis pathway (Silva et al., 2020), suggesting that *P. brasiliensis* blocks siderophore biosynthesis and can explore those molecules in the environment to scavenge iron. Silenced mutants of the *sidA* gene were obtained by antisense RNA technology, which displayed decreased siderophore biosynthesis in iron deprivation and reduced virulence to an invertebrate model. Studies have also indicated that the fungus could use both a non-classical reductive iron assimilation (RIA), comprising ferric reductases and Fe/Zn permeases, under iron-limited conditions (Bailão et al., 2015). We have demonstrated that *Paracoccidioides* spp. is able to reduce iron, and the reductase activity is linked to ferric iron uptake in *P. brasiliensis*. After reduction, the data suggest that Fe^2+^ is probably internalized through an Fe/Zn permease (Zrt). This suggestion is because *Paracoccidioides* spp. genomes do not present a ferric reductase *ftr1* homolog and the *zrt1* and *zrt2* transcripts are upregulated during iron deprivation. Of particular relevance is the fungus ability to use hemoglobin as the preferential host iron source for *Paracoccidioides* spp. To acquire hemoglobin, the fungus presents hemolytic activity and the ability to internalize the entire molecule instead of promoting the iron release extracellularly. A Glycosylphosphatidylinositol (GPL)-anchored hemoglobin receptor, Rbt5, has been described as a virulence factor (Bailão et al., 2014).

Despite the robust contribution of those studies performed by our group, there is still a need to expand the knowledge about how fungi of this genus respond to Fe depletion. Some studies that addressed *Paracoccidioides* exoproteome are available in scientific literature; however, the changes that Fe depletion induces in the exoproteome of these fungi have not been investigated yet (Vallejo et al., 2012; Weber et al., 2012; Chaves et al., 2015; Rodrigues et al., 2018). The present work addresses the changes triggered by Fe deprivation in the exoproteome of *P. brasiliensis* and lists and explores new proteins involved in that context.

## Materials and Methods

### Ethics Statement

Animal experiments were approved by the Ethics Committee on the use of Animal Experimentation (Federal University of Goiás, CEUA-UFG) under protocol number 018/20 following the guidelines of the Brazilian National Council for Control of Animal Experimentation.

### Strain Used and Culture Media

The *P. brasiliensis Pb*18 isolate (ATCC 32069–*Pb*18) was used in the present study. The yeast form of the fungus was maintained by cultivating it in semisolid Fava Netto medium supplemented with glucose 4% (w/v) at a temperature of 36°C (Fava Netto, 1961). To obtain *P. brasiliensis* exoproteomes under Fe depletion, cells cultured for 3 days in semisolid Fava Netto medium were inoculated in 250 ml of liquid Fava Netto medium supplemented with 4% glucose (w/v) and maintained for 72 h under constant agitation (120 rpm) at a temperature of 36°C. Then, fungal cells were washed 2 times (800 g, 5 min, 4°C) with PBS. Cell viability was verified by the trypan blue method, and 10^6^ viable cells/ml were transferred to 250 ml of chemically defined Chemically defined minimal medium modified, without Fe (Restrepo and Jiménez, 1980). The cells were kept for 48 h under constant agitation (120 rpm) at a temperature of 36°C. For the condition of Fe depletion (treatment), 50 µM of the Fe chelator bathophenanthrolinedisulfonic acid (BPS; Sigma-Aldrich, St. Louis, MO, USA) was added, since in a previously published work, cultivation in minimal medium (MMcM) of *P. brasiliensis* exposed to this chelator molarity for 48 h did not affect fungus viability (Parente et al., 2011). For the control condition, 10 µM of Fe (NH_4_)_2_(SO_4_)_2_ was added to the medium. All media and solutions were prepared with ultrapure water for Fe depletion experiments. All glassware used for the preparation of media and solutions was previously treated with 5N HCl for 1 h and washed extensively with ultrapure water afterward, as a strategy to minimize contamination by metals, including Fe.

### Obtaining the Exoproteome of *P. brasiliensis*

To obtain *P. brasiliensis* exoproteome under Fe depletion, the strategy described by Weber et al. (2012) was followed with some modifications. The culture supernatants were collected (800 g, 15 min, 4°C), and to minimize the contamination by possible fungal cells in suspension, the supernatants were filtered through membranes of 0.22-µm pores. Subsequently, the filtered supernatants were concentrated 250 times through 10-kDa exclusion-level membranes (Amicon Ultra centrifugal filter, Millipore, Bedford, MA, USA) and washed 3 times with 50 mM NH_4_HCO_3_ buffer, pH 8.5. The entire process of concentration and handling of the samples was carried out at low temperatures (≤4°C) to minimize the activity of proteases. The samples obtained were stored at -20°C until they were used. To verify the possibility of cell lysis, we performed diagnostic PCR for the detection of genomic DNA in culture supernatants, as described by Weber et al. (2012).

### Sample Preparation for NanoUPLC-MS^E^

Exoproteomes obtained were quantified using the Bradford method (Bradford, 1976). Afterward, 150 µg of proteins from each biological replica (three of each condition) were individually prepared to be subjected to high-resolution liquid chromatography, on a nanoscale, coupled to mass spectrometry with independent data acquisition (nanoUPLC-MS^E^), as previously described (Murad et al., 2011). Initially, 10 µl of 50 mM NH_4_HCO_3_, pH 8.5, was added to the samples. Then, as a surfactant, 75 µl of a 0.2% (w/v) RapiGEST™ solution (Waters, USA) was added, and the mixture was incubated at 80°C for 15 min. After this incubation period, 2.5 µl of 100 mM Dithiothreitol (DTT), a disulfide bridge-reducing agent, was added and a new incubation, at 60°C, for 30 min was performed. At the end of the incubation period, when the samples reached room temperature, we added 2.5 µl of 300 mM iodoacetamide, an alkylating agent, and the mixture remained at rest for 30 min at room temperature, protected from light. Then, the samples were submitted to tryptic digestion. For that, 30 µl of a 0.05-µg/µl trypsin solution (Promega, USA) was added and incubation at 37°C for 16 h was performed. Subsequently, for precipitation of the surfactant, 30 µl of 5% trifluoroacetic acid (v/v) was added and incubation at 37°C was carried out for another 90 min. Then, the samples were centrifuged at 13,000 g for 30 min, at 4°C, and the supernatants were transferred to new tubes. The centrifugation process was repeated until there was no more formation of precipitate. The samples were concentrated in a vacuum. The peptides obtained from each sample were resuspended in 80 µl of a solution containing 20 mM ammonium formiate at pH 10 and 200 fmol of Rabbit Phosphorylase B (PHB; Waters Corporation, Manchester, UK) (MassPREPTM protein). PHB was used as an internal standard for the quantification of the obtained peptides.

### High-Performance Liquid Chromatography at Nanoscale Coupled to Mass Spectrometry

The samples that underwent the tryptic digestion treatment described in the previous session were subjected to high-resolution liquid chromatography, on nanoscale, using the ACQUITY UPLC^®^ M-Class system (Waters Corporation, USA). Peptide fractionation was performed in a reverse-phase pre-column XBridge^®^ Peptide 5 µm BEH130 C18 300 µm × 50 mm (Waters, USA), a system that was maintained in a flow of 0.5 μl/min with an initial condition of acetonitrile (ACN) of 3% (v/v), representing the first dimension. The peptides were subjected to 5 fractionations (F1-F5) through different linear gradients of ACN concentrations (F1, 11.4%; F2, 14.7%; F3, 17.4%; F4-20, 7%; and F5, 50%). To perform the second dimension, each fraction was eluted in a Trap trapping column, 2D Symmetry^®^ 5 µm BEH100 C18, 180 µm × 20 mm (Waters, USA) and passed through an analytical column separation Peptide CSH™ BEH130 C18 1.7 µm, 100 µm × 100 mm (Waters, USA), in a flow of 0.4 μl/min at 40°C. The human [Glu1]-Fibronopeptide B protein (GFP; Sigma-Aldrich, USA) was used for mass calibration, which was measured every 30 s and in a constant flow of 0.5 µl/min. GFP was used at a concentration of 200 fmol. The peptides were identified and quantified by a Synapt G1 MSTM mass spectrometer (Waters, USA) equipped with a NanoElectronSpray source and two mass analyzers [a first quadrupole and the second flight time (TOF) operating in V mode], operating in MSE mode, which switches between low energy (6V) and high energy (40V) in each acquisition mode every 0.4 s. Adding the biological replicates, each condition went through 8 experimental replicates.

### Spectra Processing and Proteomic Analysis

After nanoUPLC-MSE, data processing was performed using ProteinLynx Global Server version 3.0.2 (PLGS) software (Waters, Manchester, UK), which allowed the determination of the exact mass retention time (EMRT) of the peptides and their molecular weight through the mass/charge ratio (m/z). For the identification of peptides, the spectra obtained (together with reverse sequences) were compared with sequences from the database of *P. brasiliensis* (https://www.uniprot.org/uniprot/?query=paracoccidioides+brasiliensis+strain+pb18&sort=score). Protein identification criteria included the following: (i) detection of at least two ions per fragment of peptides, (ii) five by protein fragments, (iii) determination of at least one peptide per protein, (iv) detection rate of false positive at most 4%, (v) cysteine carbamidomethylation, (vi) methionine oxidation, (vii) serine, threonine and tyrosine phosphorylation, (viii) and a trypsin lost cleavage site was allowed. Microsoft Office Excel (Microsoft^®^, USA) was used for the management of tables and the generation of graphs. In the subsequent analyses, proteins present in at least two of the three experimental replicates of each biological replicate were included. Proteins present in at least two of the three biological replicates were subjected to differential expression analysis. For this purpose, initially, the proteins that presented the lowest variance coefficient and that were detected in all replicates were used for intensity normalization. Afterward, the Expression Algorithm (Expression^E^), which is part of the PLGS software (Geromanos et al., 2009), was used for the analysis of differential expression. Proteins were considered regulated with differences (fold change) ± 2.0 between the quantification in the extract obtained in the depletion of Fe × presence of Fe. Homology search for hypothetical proteins were obtained through the online tool BLASTp (Basic Local Alignment Search Tool–https://blast.ncbi.nlm.nih.gov/Blast.cgi?PAGE=Proteins). Information on molecular function, biological processes, and subcellular location of the identified proteins was obtained from the *Paracoccidioides* database (available at http://paracoccidioides.com/). The protein sequences were subjected to additional *in silico* analysis to check for the presence of signal peptide using the online tool SignalP 4.1 Server (available at http://www.cbs.dtu.dk/services/SignalP-4.1/). For the prediction of proteins secreted by non-classical pathways, the online tool SecretomeP 2.0 (available at http://www.cbs.dtu.dk/services/SecretomeP/) was used.

### RNA Extraction and Quantitative Real-Time PCR (RT-qPCR)

After incubation for 6 and 24 h in MMcM supplemented with 50 µM of BPS or 10 µM of Fe(NH_4_)_2_(SO_4_)_2_, the yeast cells were collected and total RNA extraction was accomplished using TRIzol (TRI Reagent, Sigma-Aldrich, St. Louis, MO, USA) and mechanical cell rupture (Mini-Beadbeater–Biospec Products Inc., Bartlesville, OK, USA). SuperScript III First-Strand Synthesis SuperMix (Invitrogen, Life Technologies) was used to obtain the cDNAs that were submitted to RT-qPCR in the QuantStudio™ 5 real-time PCR System (Applied Biosystems Inc.) using SYBR Green PCR Master Mix (Applied Biosystems, Foster City, CA). The reaction was performed in triplicate for each cDNA. Normalization used the gene encoding the *L34* protein (PADG_04085). The standard curve method for relative quantification was used for calculating the relative expression levels of transcripts of interest. Standard curve was obtained using an aliquot from each cDNA sample. Statistical analysis was based on the Student’s t-test, and p values ≤0.05 were considered statistically significant. Primers used in RT-qPCR are shown in **Table S1**.

### Recombinant Cyb5 Expression in *Escherichia coli*, Protein Purification, and Polyclonal Antibodies

Total RNA was extracted from fungal yeast cells using the TRIzol reagent (TRI Reagent^®^, Sigma-Aldrich, St. Louis, MO, USA) and mechanical cell rupture (MiniBeadbeater—BioSpec Products), as described by the manufacturer’s protocol. From the extracted RNA, cDNA was synthetized following the manufacturer recommendation of the SuperScript^®^ Reverse Transcriptase Kit (Invitrogen™, Waltham, MA, USA). The cDNA was used to amplify the *cyb5* gene (PADG_03559) using the polymerase High Fidelity (Invitrogen™, Waltham, MA, USA). The cDNA product obtained by RT-PCR was cloned into the expression vector pET-32a. Bacterial cells, strain *Escherichia coli* C43, harboring the recombinant plasmid were grown in Luria–Bertani (LB) medium supplemented with 100 µg/ml ampicillin (w/v) under agitation at 37°C until the optical density (OD) reached an absorbance of 0.6 at a wavelength of 600 nm. The reagent isopropyl-β-D-thiogalactopyranoside (IPTG) was added to the growing culture to a final concentration of 0.1 mM. Bacterial cells were harvested by centrifugation at 10,000 × g for 10 min after 16 h of incubation at 15°C and resuspended in phosphate buffered saline (PBS) 1×. The recombinant Cyb5 protein fused to Trx-His-Tag was used to produce polyclonal antibodies in 4 BALB/c male mice aged 6–8 weeks. The fusion protein was removed from Sodium Dodecyl Sulfate Polyacrylamide Gel Electrophoresis gels and injected into mice along with Freund’s adjuvant three times at intervals of 15 days. Serum containing polyclonal antibodies was collected and stored at −20°C. The protein was produced in inclusion bodies and was solubilized using 50 µl of a 20% (w/v) N-lauroylsarcosine sodium salt (Sigma Aldrich, Missouri, KS, USA) solution for 5 ml of bacterial extracts and sonicated (5 times, 10 min). SDS-PAGE analysis showed the protein in the soluble fraction, and then the protein was purified by a nickel resin chromatography system (Qiagen Inc., Germantown, MD, USA).

### Western Blotting

Proteins in SDS-PAGE were transferred to the nitrocellulose membrane that was then incubated with polyclonal anti-*Pb*18Cyb5 at 1:250 dilution for 2 h at room temperature. After washing, the membranes were incubated with peroxidase-coupled mouse anti-IgG secondary antibody (1:1,000 dilution). The reaction was revealed by chemiluminescence with the ECL Western Blotting Analysis System (GE Healthcare). Negative control was obtained with pre-immune mouse serum (1:250 dilution). Reaction was developed in a chemiluminescent imager (Amersham Imager 600, GE Healthcare).

### Dot-Blot Analysis

Nitrocellulose membrane containing 30 μg of FeSO_4_ and BPS exoproteome extracts was incubated with anti-*Pb*18Cyb5 polyclonal antibodies (diluted 1:500) or pre-immune sera (diluted 1:1,000). Antibody anti-mouse IgG coupled to peroxidase (diluted 1:1,000) from ECL Western Blotting Analysis System (GE Healthcare) was used as secondary antibody. Reaction was developed in a chemiluminescent imager (Amersham Imager 600, GE Healthcare).

### Immunofluorescence Assays

For immunofluorescence, 10^6^ yeast cells/ml were fixed in ice-cold pure methanol for 3 h at 20°C. Subsequently, cells were incubated for 30 min at room temperature in the dark in blocking buffer containing 3% (w/v) bovine serum albumin (BSA-Sigma) and 0.2% (v/v) Tween 20 in PBS, followed by incubation with the primary anti-*Pb*18Cyb5 polyclonal antibodies at 1:250 dilution, for 1 h. Subsequently, it was added with fluorescein isothiocyanate-labeled mouse secondary antibody-FITC (Sigma) at 1:750 dilution for 1 h. Cells were washed three times with PBS. Images were taken in bright field and at 450–490 nm for visualization of FITC fluorophore using the Axio Scope A1 fluorescence microscope. Digital images were acquired using AxionVision software (Carl Zeiss AG, Germany).

### 3D Structure Prediction *via* Molecular Modeling

The three-dimensional structure of Cyb5 heme-binding protein was predicted by the I-TASSER (Iterative Threading Assembly Refinement) server (Zhang, 2008; Yang et al., 2015). The structural modeling relies on templates of homologous proteins available on the PDB (protein data bank) database (Berman, 2000). The I-TASSER server uses Monte Carlo simulations to cluster homologous fragments together (Swendsen and Wang, 1986). The determination of the best protein conformation starts *via* prediction of the target secondary structure by PSSpred (Protein Secondary Structure Prediction) and the identification of similar templates by LOMETS (Local Meta-Threading-Server) (Wu and Zhang, 2007). The distribution of templates into clusters allows the classification of such sequences according to topology and stability through SPICKER (Zhang and Skolnick, 2004) in order to predict structures that are more likely to be similar to the native condition of the protein target. Eventually, the best ranked structures are submitted to molecular dynamics, and the biological function is predicted by COACH (Yang et al., 2013). The quality of the final structure was assessed through MolProbity (Williams et al., 2018).

### Prediction of Iron-Binding Sites in Cytochrome b5

The prediction of the iron-binding sites in the Cyb5 structure was performed by the fragment transformation method (FTM). This approach compares the structure of the target with experimentally determined metal iron-binding proteins available in the PDB. The server MIB (metal ion binding) compares iron-binding site templates and takes into consideration residues within at least 3.5 Å from the iron center. FTM (Lu et al., 2006) performs structural alignments of fragments from the target protein and the iron-binding template. A score is attributed to the amino acids in the target protein based on similarity and according to a BLOSUM substitution matrix (Henikoff and Henikoff, 1992) and the root mean square deviation (RMSD) of the alignments. Amino acids with a score higher than the cutoff value (1.0 and 95% accuracy) are prone to bind to iron.

### Spectrophotometric Analysis of the Interaction Between Recombinant Cytochrome b5 and Inorganic Iron

The binding capacity of Cyb5 to iron was investigated experimentally. Spectrophotometric analyses were performed as described (Oliveira et al., 2020). Briefly, the purified Cyb5 protein treated or not previously with reducing solution (sodium hydrosulfite at 100 μM, 1 h at room temperature; Sigma-Aldrich, USA, Ref. No. 71699) was subjected to incubation in ferrous sulfate at 100 μM (Sigma-Aldrich) for 1 h at room temperature. Then, samples and controls were deposited in a 96-well plate and subjected to absorbance analysis on a SpectraMax^®^ Paradigm^®^ Multi-Mode Detection Platform (Molecular Devices, Austria). The scan was performed between the wavelengths of 230–700 nm.

## Results

### Exoproteome Analysis of *P. brasiliensis* Submitted to Fe Deprivation

**Figure 1:**
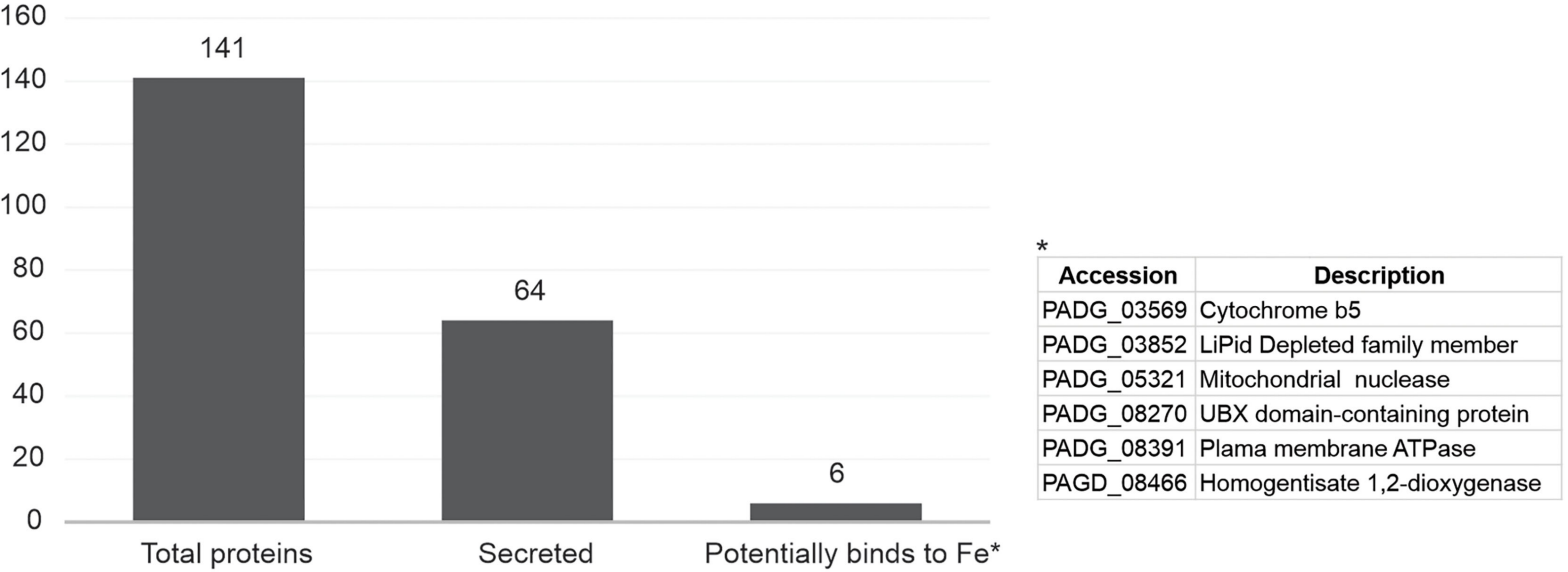
Summary of proteomic findings. *P. brasiliensis* yeast cells were subjected to Fe deprivation for 48 h, and fungal secreted proteins were identified by nanoUPCL-MS^E^. A total of 141 proteins were identified. To increase the analysis stringency, the identified proteins were submitted to analysis of secretion prediction by online tools and data available in the literature, which resulted in the number of 64 proteins. Of those 64 proteins, 6 have potential ability to bind to iron.

To verify the possible changes that Fe deprivation can promote in *P. brasiliensis* exoproteome, the fungus was grown in depletion or presence of the metal. Culture supernatants containing the proteins secreted by the fungus were collected and, after tryptic digestion, subjected to nanoUPLC-MS^E^. As an indirect way of verifying the presence of cytoplasmic contaminants (cell lysis), we checked by diagnostic PCR the presence of genomic DNA in the culture supernatants (Weber et al., 2012). According to **Supplementary Figure S1**, there was no amplification of genomic DNA from samples of culture supernatants. Thus, we carried out the analysis. In agreement with **Figure 1** and **Supplementary Table S2**, a total of 141 proteins were identified, of which, considering the fold change of 2.0, 93 (66%) were positively regulated (**Supplementary Table S3**). Prediction was made for protein secretion by a classical pathway (signal peptide dependent), secretion by non-classical pathways, or evidence of secretion in the *Paracoccidioides* literature (Vallejo et al., 2012; Weber et al., 2012; Chaves et al., 2015; Rodrigues et al., 2018; Moreira et al., 2020). As shown in **Figure 1**, 64 proteins (45.4%) were predicted to be secreted. Information on molecular function, classic biological processes, and classic subcellular location of the identified proteins is available in **Supplementary Tables S2, S3**.

Among the positively regulated proteins, a virulence factor of *P. brasiliensis* previously described was identified as serine proteinase (PADG_07422), in addition to adhesins such as enolase (PADG_04059), dihydrolipoil dehydrogenase (PADG_06494), and translation elongation factor Tu (PADG_01949) (Borges et al., 2005; Donofrio et al., 2009; Nogueira et al., 2010; Parente et al., 2010; Marcos et al., 2012; Marcos et al., 2016; Landgraf et al., 2017; Pigosso et al., 2017). Regarding the fungus response to Fe depletion, a gamma-glutamyl transpeptidase was identified (PADG_01479) 2.4 times more expressed. There were proteins identified only in the condition of depletion of Fe: homogentisate 1,2-dioxigenase (PADG_08466), plasma membrane ATPase (PADG_08391), UBX domain-containing protein (PADG_08270), mitochondrial nuclease (PADG_05321), LiPid-depleted family member (PADG_03852), previously predicted as Fe ligands (Tristão et al., 2015), and surprisingly, Cyb5 (PADG_03559), positively regulated 20.4 times.

### Iron Deprivation Promotes Upregulation of Transcripts of *Paracoccidioides Cyb5* and *Ggt2* Genes

**Figure 2:**
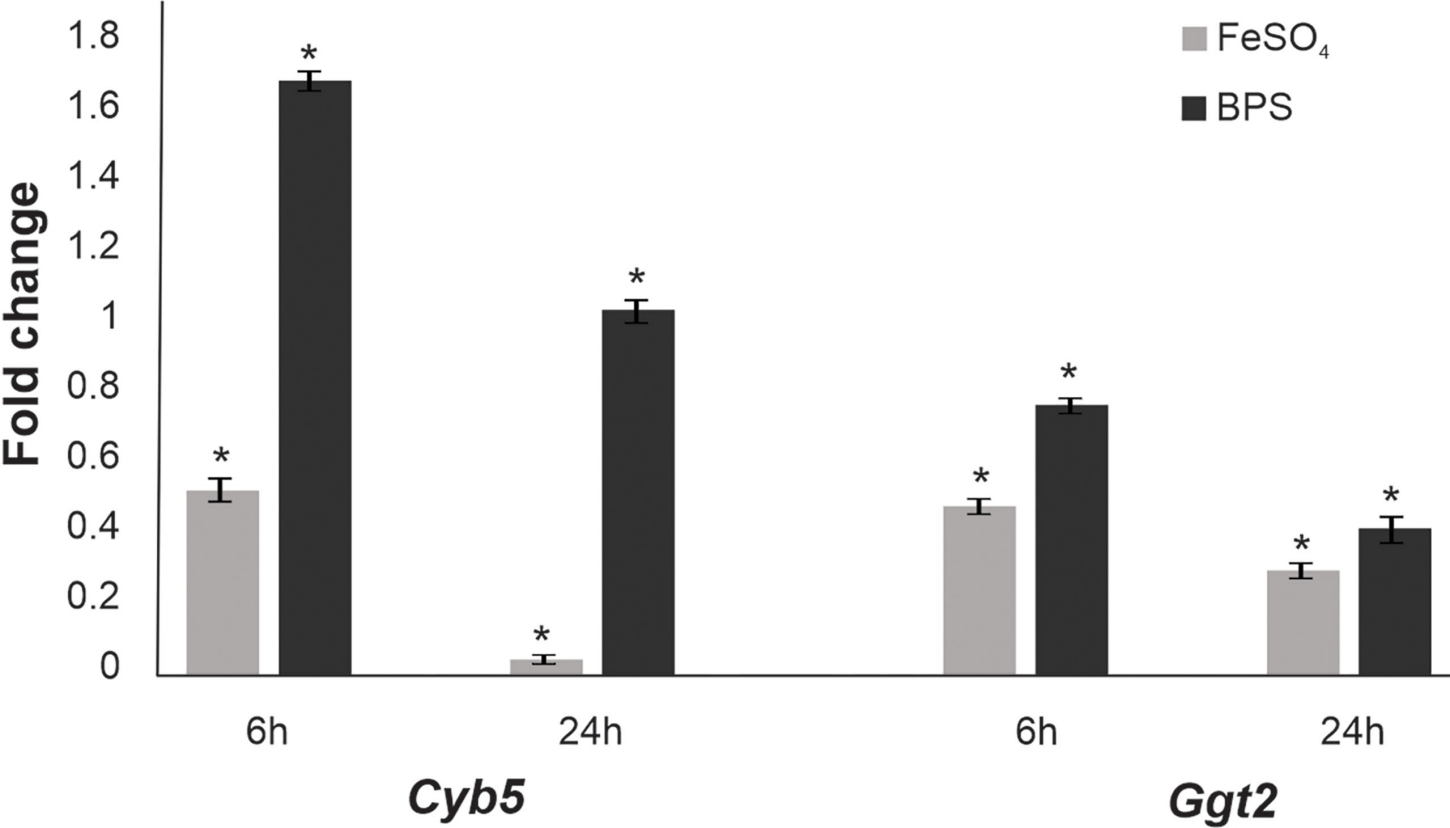
Expression quantification of selected genes in *Paracoccidioides brasiliensis* by RT-qPCR. Quantitative RT-PCR data showing the transcript levels of cytochrome b5 (*Cyb5*) and gamma-glutamyl transpeptidase (*Ggt2*) in the presence and absence of iron at 6 and 24 h. The data were normalized using the gene encoding the 60S ribosomal protein L34 as the endogenous control and are presented as relative expression to the control. Data are expressed as the mean ± standard deviation of the triplicates of independent experiments. Student’s t-test was used for statistical comparisons. Error bars represent the standard deviation of three biological replicates, and * represents p ≤ 0.05.

In order to validate the results obtained in proteomics, we performed the analysis of *cyb5* (PADG_03559) and *ggt2* (PADG_01479) gene transcripts, since the resulting proteins were identified as upregulated in the imposed Fe deprivation condition. For that, we used RT-qPCR, and, according to **Figure 2**, it is possible to verify that positive regulation of the products of these genes occurs. These data corroborate the proteomic findings. Additionally, it is possible to notice that there is higher gene expression at the 6-h point, which can be associated with the flow of gene information and the consequent accumulation of Cyb5 and Ggt2 proteins at the 48-h time point used for proteomics.

### Cytochrome b5 Is Secreted in Response to Iron Deprivation

**Figure 3:**
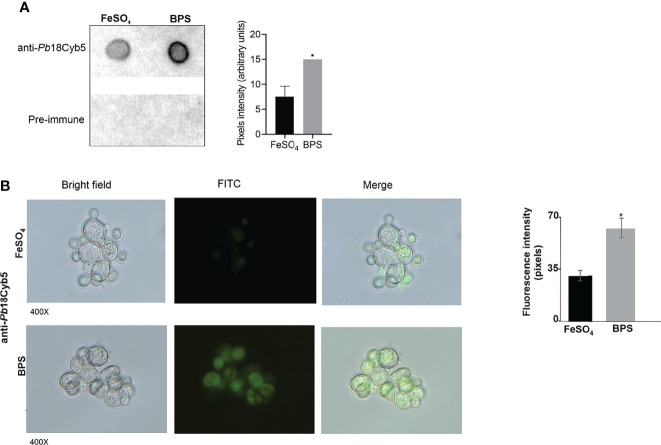
Analyzing the expression dynamics of *Paracoccidioides* Cyb5 in iron deprivation. **(A)** Dot-blot analysis. Nitrocellulose membranes containing exoproteomes (FeSO_4_ and BPS) were incubated with anti-*Pb*18Cyb5 (1:500) polyclonal antibodies or pre-immune sera (1:1,000). Pixel intensity was measured by densitometric analysis of immunoblotting dots using ImageJ software. Statistics analysis was performed through Student’s t-test. * Represents p ≤ 0.05.**(B)** Fluorescence microscopy of *P. brasiliensis* (*Pb*18) cells cultured in the presence or absence of iron for 24 h and subsequently incubated with primary antibody anti-Cyb5 and later with the secondary antibody anti-mouse IgG labeled with fluorescein isothiocyanate (FITC; Sigma). The data for fluorescence intensity evaluation were obtained through the AxioVision Software (Carl Zeiss). The values of fluorescence intensity (in pixels) and the standard error of each analysis were used to plot the graph. Data are expressed as mean ± standard error (represented using error bars); * represents p ≤ 0.05. One hundred cells of each condition were evaluated. All representative images in panel **B** were magnified ×400.

The fact that the Cyb5 protein was upregulated more than 20 times in the *P. brasiliensis* exoproteome led us to further investigate the secretion dynamics of this protein. Therefore, recombinant Cyb5 was produced (**Supplementary Figure S2A**) and injected into mice to obtain polyclonal serum that specifically recognized the recombinant protein (**Supplementary Figure S2B**). Then, we verified whether the polyclonal serum obtained was able to recognize the Cyb5 protein in *P. brasiliensis* exoproteome. Therefore, we performed dot-blot analysis and verified that Cyb5 is recognized in the exoproteome of *P. brasiliensis*, with higher amount in the exoproteome obtained from iron deprivation condition, corroborating the proteomic findings (**Figure 3A**). Afterward, by indirect immunofluorescence assays, it was possible to verify that in non-permeabilized cells of *P. brasiliensis*, Cyb5 was present on the surface and presented a higher fluorescence in cells exposed to Fe deprivation, as shown in **Figure 3B**. All these findings confirm proteomics results and place Cyb5 as a molecule potentially involved in the response of *P. brasiliensis* to Fe deprivation.

### *Pb*Cyb5 Is an Fe-Binding Protein

**Figure 4:**
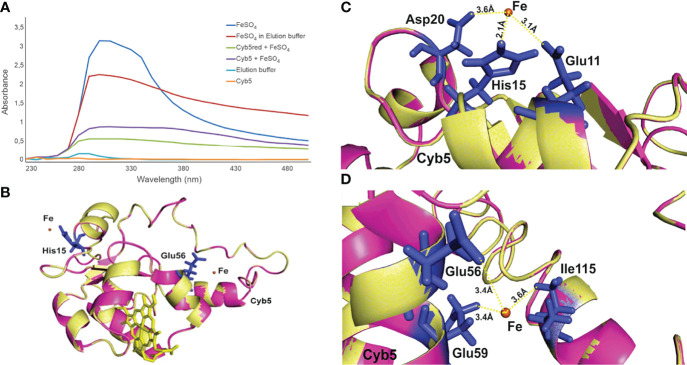
Cyb5 of *Paracoccidioides brasiliensis* is an Fe-binding protein. **(A)** Absorbance spectra of the purified recombinant Cyb5 protein in a lower oxidation state (green—sample previously treated with a reducing agent) and higher oxidation (purple). Controls: ferrous sulfate solution (dark blue), ferrous sulfate prepared in elution buffer (red), elution buffer (light blue), and purified recombinant Cb5 (orange). **(B)** The structural alignment of Cyb5 with iron-binding motifs resulted in two sites of iron binding for this protein with significant scores. Residues interacting with the iron ion are shown as sticks and labeled as His for histidine and Glu for glutamate. These are the amino acid residues less distant from the iron centers. Iron is shown as a brown sphere. **(C)** The structural alignment showed that His15 binds to iron in the distance of 2.1 Å, reaching a score above the threshold. The same residue may interact with iron more than once. Glutamic acid 11 (Glu11) and aspartic acid 20 (Asp20) bind to iron in the distance of 3.1 and 3.6 Å, respectively. **(D)** The structural alignment showed that the residues Glu56 and Glu59 bind to iron within the distance of 3.4 **(A)** Isoleucine 115 (Ile115) binds to iron with a lower score but still above the threshold. Bonds to the iron are shown as dotted yellow lines, with bond distances indicated next to the lines.

The fact that Cyb5 is a relatively small heme-containing protein and the high regulation shown in cells submitted to Fe deprivation placed this protein as a possible mediator of the fungal response to Fe deprivation. We then investigated the ability of recombinant Cyb5 to bind Fe by spectroscopic analysis; the results identified the possible event at least *in vitro* (**Figure 4A**). To refine our findings, we performed molecular modeling analyses of the Cyb5 and iron interaction. The protein from the PDB that has the closest structural similarity to Cyb5 from *P. brasiliensis* with the highest TM score (0.712) was the human Cyb5 under the PDB accession number 2I96. We identified two potential iron-binding sites in the Cyb5 protein with scores higher than 1.0 (**Figure 4B**). The amino acid residue His15 scored 1.773 and Glu56 scored 1.225. The two-colored regions in **Figure 4** represent the structural alignment performed between the target protein Cyb5 and the iron-binding site templates. The interaction distance between His residue and iron is 2.1 Å (**Figure 4C**) and between Glu is 3.4 Å (**Figure 4D**). The higher score achieved for the His residue is related to a smaller distance between this amino acid and the iron ion. It is important to highlight that the interaction sites of Cyb5 with Fe occur in regions different from where the heme group binds, which suggests that the interaction of Cyb5 with Fe does not depend on the heme group. In addition, other residues bind to iron with a lower score but above the threshold, showing that several residues contribute to the free energy of binding to the metal ion. Glutamic acid 11 (Glu11) and aspartic acid 20 (Asp20) bind to iron in the distance of 3.1 and 3.6 Å, respectively (**Figure 4C**), and isoleucine 115 (Ile115) binds to iron in the distance of 3.6 Å (**Figure 4D**).

## Discussion

Previous studies have demonstrated the importance of the response of *Paracoccidioides* spp. to depletion of Fe for the pathogenicity of fungi of this genus, since their exposure to conditions that mimic those found in the host induces the expression of genes related to high-affinity and -specificity mechanisms for Fe uptake (Bailão et al., 2006; Bailão et al., 2007; Parente et al., 2011; Bailão et al., 2014; Kuznets et al., 2014; Bailão et al., 2015). Large-scale proteomic analyses have contributed positively in recent years to the understanding of the biology of *Paracoccidioides* spp. Studies with a similar approach enlisted virulence factors and metabolic profiles in response to several stressful conditions, including Fe depletion (Parente et al., 2011). Despite the biological relevance of the fungal secretory response to stressful conditions, as far as we know, there are no published studies that have evaluated the effect of Fe depletion on the exoproteome of pathogenic fungi. The only work available in the literature with a similar approach is that of Sorgo et al. (2013), who performed an analysis of the cell wall proteome of *Candida albicans* after exposure of the fungus to Fe depletion, whose results pointed to the positive regulation of proteins related to high-affinity mechanisms for capturing the metal, such as Als3, Rbt5, and Pga7 (Sorgo et al., 2013). The scarcity of studies using this approach points to the relevance of the present study, which can be extended beyond the genus *Paracoccidioides*.

Of the 64 proteins identified in the present work, 36 were also identified in the work performed by Vallejo et al. (2012), who studied the protein content of extracellular vesicles of *Paracoccidioides*, structures whose biology and impact on host–pathogen interaction have been elucidated in recent years (Vallejo et al., 2012; da Silva et al., 2016; Peres da Silva et al., 2019; Baltazar et al., 2021). Finding proteins from these structures in our work confirms that our sample enrichment strategy was effective. It was observed that Fe depletion promoted the positive regulation of virulence factors already described for *Paracoccidioides* spp. (Gonzalez and Hernandez, 2016). Among the virulence factors identified, it is worth mentioning serine proteinase (PADG_07422), a virulence factor described for *Paracoccidioides* spp. (Parente et al., 2010). In an intranasal murine infection model, this enzyme was secreted in the lung tissue and therefore listed as a potential participant in the pathogenesis process (Pigosso et al., 2017). Of relevance, the protein is also a surface molecule, promoting the fungus interaction with macrophages (Tomazett et al., 2019). It should be noted that in a previous study, serine proteinase showed positive regulation at the transcriptional level when the fungus was treated with human plasma, which hypothetically occurred due to the depletion of Fe experienced by the fungus under suh condition (Bailão et al., 2007). This hypothesis was built based on the information that *Bacillus subtilis* employs a serine proteinase for the proteolysis of transferrin, a protein that transports Fe in mammals. The Fe released in the process allows the uptake of the metal mediated by bacterial siderophores (Park et al., 2006). The positive regulation of serine proteinase after Fe depletion in the present study corroborates this hypothesis.

In the present work, it was also possible to verify the positive regulation of *Paracoccidioides* spp. adhesins previously described. Enolase (PADG_04059) was 2.2 times more expressed in the Fe depletion condition. This enzyme classically known as a participant in glycolysis was also described on the cell surface of *P. brasiliensis* (Oliveira et al., 2012). Enolase has adhesin properties, since it is able to bind to the cell matrix components such as fibronectin and plasminogen and promotes the adhesion of the fungus to A549 epithelial cells and murine macrophages (Donofrio et al., 2009; Nogueira et al., 2010; Oliveira et al., 2012). The enzyme dihydrolipoyl dehydrogenase (PADG_06494) was described in a relatively recent work as an exoantigen of *P. brasiliensis*. The stimulation of macrophages with the recombinant protein increased their phagocytic and microbicidal activity, which positioned this protein as possibly related to the interaction of the fungus with the host (Landgraf et al., 2017). Another positively regulated adhesin identified in the present study was the Tu translation elongation factor (PADG_01949), described as a fibronectin and plasminogen ligand and participant in the interaction of *P. brasiliensis* with pneumocytes (Marcos et al., 2016). It should be noted that despite previous works that evaluated the exoproteome of *Paracoccidioides* spp., this is the first time that this protein has been identified in this subproteome, which reiterates iron depletion as a modulating condition of the fungus exoproteome. It is worth mentioning the protein homogentisate 1,2-dioxigenase (PADG_08466), which was identified only in the condition of Fe depletion. Previous work points out that this protein is also a plasminogen ligand putatively involved in the process of adhesion, invasion, and spread of the fungus during infection (Chaves et al., 2015).

Glutathione-dependent ferric reductase activity has been reported by a previous study in culture supernatants of *Blastomyces dermatitidis*, *Sporothrix schenckii*, *Histoplasma capsulatum*, and *P. brasiliensis* (Zarnowski and Woods, 2005). Later work demonstrated that in *H. capsulatum*, this activity is dependent on a gamma-glutamyl transpeptidase (Zarnowski et al., 2008). The enzyme has been shown to act on glutathione to generate the dipeptide cysteinylglycine, which has a strong reducing power in a pH-dependent process (Zarnowski et al., 2008). *P. brasiliensis* presents two GGTs: PADG_07986 (Ggt1) and PADG_01479 (in this work called Ggt2). Homology studies and transcriptional analyses have identified Ggt1 as the secreted enzyme active in the reduction of Fe^3+^ (Silva et al., 2011; Bailão et al., 2012; Bailão et al., 2015). Previous studies identified Ggt2 in the exoproteome of *P. brasiliensis* and *P. lutzii* (Vallejo et al., 2012; Weber et al., 2012). In the present work, we identified the positive regulation of gamma-glutamyl transpeptidase (PADG_01479) Ggt2 in the *P. brasiliensis* exoproteome subjected to metal depletion, corroborating the literature and providing new information about the enzyme.

A very relevant finding in the present work was the upregulation of *Pb*Cyb5, secreted in response to Fe deprivation. Cyb5 and related enzymes are involved in multiple cellular processes, including sterol biosynthesis, maintenance of cell membranes, and azole resistance (Misslinger et al., 2017; Zhang et al., 2021). Cyb5 is also related to the regulation of Fe homeostasis in *S. cerevisiae* (Dap1) and *A. fumigatus* (CybE) (Craven et al., 2007; Misslinger et al., 2017). The data obtained in these works seem to conflict with the ones we obtained because, instead of upregulation, the authors verified another regulation process. It should be noted that these studies focused on the intracellular level, and therefore, comparing them with exoproteome results is not an obvious task.

To the best of our knowledge, this is the first time that Cyb5 has been described in a fungal exoproteome in response to Fe deprivation. Cyb5 is able to bind inorganic iron, as shown in **Figure 4A**. By bioinformatics analyses, we verified that this protein presents a similarity to the canonical iron-binding site of transferrin, where a His residue is linked to iron in an alpha-helix domain (Baker and Baker, 2012). Histidine residues are also present as an iron-binding site in cysteine dioxygenase, which is active in cysteine thiol oxidation (Simmons et al., 2006) and as iron-detoxifying membrane transporter from pathogens (Sharma et al., 2021). Glutamate (Glu) has been identified in certain iron-binding motifs (Sharma et al., 2021), such as in membrane transporters and in iron-binding adhesins that regulate biofilm formation in bacteria (Jiang et al., 2020). The higher score achieved for the His residue is related to a smaller distance between this amino acid and the iron ion. In membrane transporters, the distance between His and Glu to the iron ion is in the range of 2.0 Å (Jiang et al., 2020), and in iron transport proteins, the His residue is also 2.0 Å from the metal ion (Abdizadeh et al., 2017).

The findings of this work demonstrate that the *Paracoccidioides* exoproteome is dynamic and that it is modulated in response to stressful conditions, such as Fe deprivation. Upregulation of several virulence factors may be linked to the predictive adaptation hypothesis, since Fe deprivation is a condition imposed by the host in association with a myriad of other stressors (Brunke and Hube, 2014). Additionally, we identified Cyb5 as a new molecule of *Paracoccidioides* spp. related to the fungal response to Fe deprivation. The current world scenario presents fungi with increasing resistance to antifungal agents and the absence of new compounds approved to treat mycoses. Studying the host–pathogen interaction interface is key to bioprospecting new antifungal targets. Since Fe uptake is an essential attribute for the infective success of pathogens, listing new molecules that act in the process is a promising strategy to list new targets for antifungal compounds. It is now necessary to investigate which are the interaction partners of Cyb5 and whether Cyb5 is sufficient to promote the availability of Fe to the fungus.

## Data Availability Statement

The datasets presented in this study can be found in online repositories. The names of the repository/repositories and accession number(s) can be found below: http://www.peptideatlas.org/, PASS01753.

## Ethics Statement

The animal study was reviewed and approved by the Comissão de ética no uso de animais (CEUA) Universidade Federal de Goiás.

## Author Contributions

CS, AS, LP, and LS conceived and designed the experiments. AS, LP, LS, IG, JP, MO, and KS performed the experiments. KS and MP performed the molecular dynamics analyses. CS and MP contributed reagents and materials. CS, AS, LP, LS and KS wrote the article. All authors have read and agreed to the published version of the article.

## Funding

Funding was provided by Fundação de Amparo à Pesquisa do Estado de Goiás, Institute Nacional of Thecnology of Host Pathogen Interaction (INCT-IPH), and Conselho Nacional de Desenvolvimento Científico e Tecnológico (CNPq) process number 302085/2019-0.

## Conflict of Interest

The authors declare that the research was conducted in the absence of any commercial or financial relationships that could be construed as a potential conflict of interest.

## Publisher’s Note

All claims expressed in this article are solely those of the authors and do not necessarily represent those of their affiliated organizations, or those of the publisher, the editors and the reviewers. Any product that may be evaluated in this article, or claim that may be made by its manufacturer, is not guaranteed or endorsed by the publisher.
