# Supplementary material for: Iron Deprivation Modulates the Exoproteome in Paracoccidioides brasiliensis
Source: Front Cell Infect Microbiol. 2022 Jun 3;12:903070. doi: 10.3389/fcimb.2022.903070 (PMC9205457; doi:10.3389/fcimb.2022.903070)
Supplement: Supplementary file 1 [file DataSheet_1.pdf]

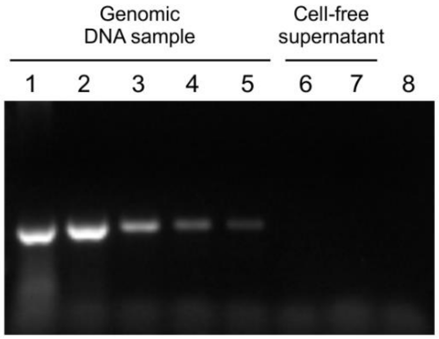

Supplementary Figure 1. Validation of the extracellular protein extraction method. Sensitivity to the formamidase gene was accessed through PCR using *P. brasiliensis* genomic DNA as a template (at five dilutions: 50 ng to 1 pg). Lanes: 1 – 50 ng; 2 – 5 ng; 3 – 50 pg; 4 – 5 pg; 5 – 1 pg; 6 – Culture supernatants in BPS; 7 – Culture supernatants in $FeSO_4$; 8 – negative control. PCR amplicons were assessed with 1,5 % (w/v) agarose gel electrophoresis and stained with ethidium bromide.

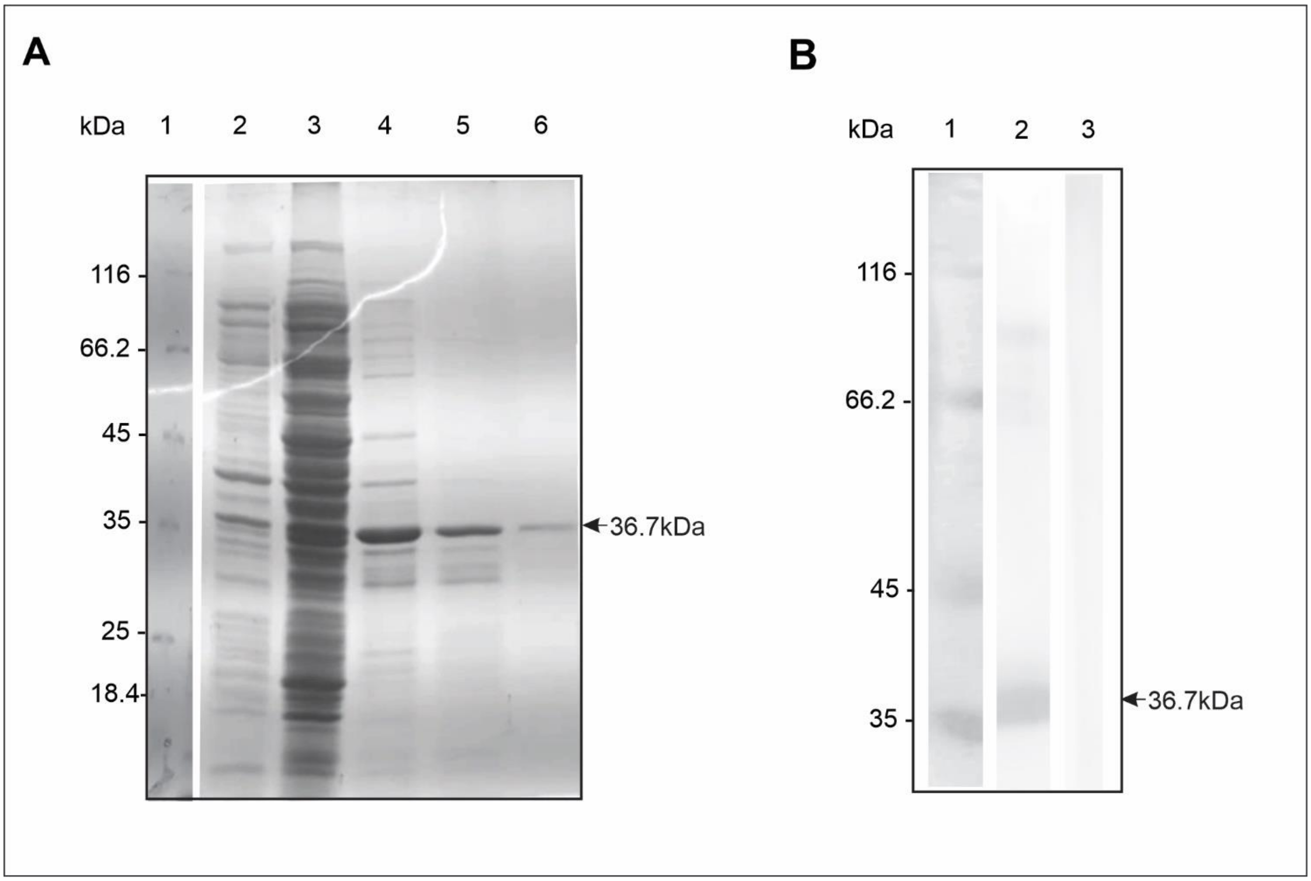

Supplementary Figure 2. Recombinant protein *Pb*18Cyb5 expression. **(A)** SDS-PAGE of Cyb5 (PADG_03559) *Escherichia coli* C43. In **1** MW: Broad Range Protein Molecular Weight Marker; **2**: *E. coli* extract before IPTG induction; **3**: induction performed by addition of Isopropyl-β-D-thiogalactopyranoside (IPTG) at a concentration of 1mM for 5 hours; **4**: recombinant protein associated with nickel resin **5:** elution of the recombinant protein with buffer containing 250 mM imidazole and **6:** purified recombinant Cyb5 after affinity-chromatography with Ni-NTA AGAROSE resin. The recombinant protein size of 36.7 kDa corresponds to 14.74 kDa of *Pb*18Cyb5 fused to 20.4 kDa of trA-His6-tag. **(B)** Immunoblotting analysis. Nitrocellulose membranes containing purified recombinant *Pb*18Cyb5 were incubated with anti-*Pb*18Cyb5 (1:250) polyclonal antibodies. Lane 1: Molecular Weight Marker (Thermo Fisher Scientific). Lane 2: purified recombinant *Pb*18Cyb5. Lane 3: Purified recombinant protein incubated with pre-immune sera (1:250).

Supplementary Table 1. Specific primers used in RT-qPCR.

Gene/ Accession number	Forward primer (5´→3´)	Reverse primer (5´→3´)
Cytochrome b5/ PADG_03559	CGATGAGCATCCTGGTGGC	CGACGAGCATGCCCTGAAG
Ggt2 / PADG_01479	CGCTGTCGCCGTTGCTGC	AATATTTCCAGCTGCACGACC
60S ribosomal protein L34/ PADG_04085	TCAATCTCTCCCGCGAATCC	AGTTGGCGATTGTTGTGCGG

Supplementary Table 2. Exoproteome of *P. brasiliensis* (*Pb*18) identified after culturing yeast cells for 48 hours at 37 ºC in MMcM liquid medium with Fe restriction (BPS 50 µM) or Fe supplementation [Fe(NH4)2(SO4)2.6H2O 10 µM]

Accession[a]	Description[b]	Score[c]	BPS:Fe Ratio[d]	SignalP ≥ 0.45[e]	SecretomeP ≥ 0.6[f]	Evidence of secretion in *Paracoccidioides* spp. literature[g]	Molecular function[h]	Weber *et al.*, 2012[i]	Vallejo *et al.*, 2012[j]	Chaves *et al.*, 2015[k]	Oliveira *et al.*, 2018[l]	Moreira *et al.*, 2020[m]	Iron Binding? Tristão *et al.*, 2015[n]
PADG_01366	NADH dehydrogenase (ubiquinone) 1 alpha subcomplex 5 •	7336	0.16	***	0.657	-	Oxidoreductase activity, acting on NAD(P)H	*	*	*	*	*	**
PADG_07891	Ubiquitin-60S ribosomal protein L40 •	5805	0.24	***	***	-	Structural constituent of ribosome	*	*	*	*	*	**
PADG_01372	Mannitol-1-phosphate 5-dehydrogenase	4989	0.25	***	0.670	-	Mannitol-1-phosphate 5-dehydrogenase activity,coenzyme binding	Yes	Yes	*	Yes	Yes	**
PADG_02022	Clathrin light chain	2841	0.29	***	0.775	-	Structural molecule activity	*	Yes	*	*	*	**
PADG_05837	E3 ubiquitin ligase complex SCF subunit sconC	14,726	0.35	***	0.706	-	DNA replication origin binding, ligase activity, ubiquitin protein ligase activity	*	Yes	*	*	*	**
PADG_08406	O-acetylhomoserine (thiol)-lyase	3946	0.39	***	***	-	Transferase activity, transferring alkyl or aryl (other than methyl) groups, lyase activity, pyridoxal phosphate binding	Yes	*	*	*	*	**
PADG_04604	Transketolase	1997	0.52	***	0.650	-	Transketolase activity	*	Yes	*	*	*	**
PADG_02056	Ribosomal protein L7/L12 •	8637	0.56	***	0.752	-	Structural constituent of ribosome	*	*	*	*	*	**
PADG_11379	60S ribosomal protein L5 •	3386	0.59	***	***	-	Structural constituent of ribosome, 5S rRNA binding, hydrolase activity	*	*	*	*	*	**
PADG_02761	Hsp75-like protein	2135	0.61	***	***	-	ATP binding	*	Yes	*	*	*	**

PADG_04730	Nascent polypeptide-associated complex subunit alpha	6273	0.71	***	0.797	-	Phosphatidylinositol-3-phosphate binding, phosphatidylinositol-4-phosphate binding, phosphatidic acid binding, phosphatidylinositol-3,5-bisphosphate binding	*	Yes	*	Yes	*	**
PADG_00674	Hypothetical protein •	2139	0.73	***	***	-	NDA	*	*	*	*	*	**
PADG_02967	Hypothetical protein	3922	0.77	***	***	-	NDA	*	Yes	*	*	*	**
PADG_07749	NAD(P)H:quinone oxidoreductase, type IV •	15,726	0.78	***	0.623	-	NAD(P)H dehydrogenase (quinone) activity,FMN binding	*	*	*	*	*	**
PADG_06906	Triosephosphate isomerase	11,326	0.84	***	0.623	-	Triose-phosphate isomerase activity	*	Yes	*	Yes	*	**
PADG_07213	Pyruvate dehydrogenase complex dihydrolipoamide acetyltransferase	2736	0.84	***	0.635	-	Dihydrolipoyllysine-residue acetyltransferase activity	*	Yes	*	*	*	**
PADG_01010	AMPK1_CBM domain-containing protein	3455	0.85	***	***	-	NDA	*	Yes	*	*	*	**
PADG_08615	Tropomyosin-1	11,021	0.87	***	0.793	-	NDA	*	Yes	*	*	*	**
PADG_03562	Chaperone DnaK	6962	0.89	0.864	***	-	ATP binding, ATPase activity	*	Yes	*	*	*	**
PADG_00183	Profilin	19,317	0.90	***	0.889	-	NDA	*	Yes	*	*	*	**
PADG_07715	Hsp90-like protein	3526	0.93	***	***	-	ATP binding	*	Yes	*	*	*	**
PADG_06992	GrpE protein homolog •	3945	0.95	***	0.846	-	Adenyl-nucleotide exchange factor activity	*	*	*	*	*	**
PADG_01363	Acyl-CoA-binding protein	11,793	1.00	***	0.873	-	Fatty-acyl-CoA binding	*	Yes	*	*	*	**
PADG_04056	14-3-3 family protein epsilon •	10,435	1.01	***	***	-	NDA	*	*	*	*	*	**
PADG_06488	Peptidyl-prolyl cis-trans isomerase D	17,659	1.02	***	***	-	Peptidyl-prolyl cis-trans isomerase activity	Yes	Yes	*	Yes	*	**
PADG_02726	Cysteine synthase	18,233	1.16	***	0.621	-	NDA	*	Yes	*	Yes	*	**
PADG_00430	Hsp7-like protein	15,295	1.17	***	***	-	ATP binding	*	Yes	*	*	*	**
PADG_05922	Glutamate carboxypeptidase	1312	1.17	***	0.630	-	Dipeptidase activity	Yes	Yes	*	Yes	Yes	**
PADG_00784	40S ribosomal protein S0 •	1850	1.20	***	***	-	Structural constituent of ribosome	*	*	*	*	*	**

PADG_00995	Ubiquitin-40S ribosomal protein S27a •	5556	1.22	***	0.834	-	Structural constituent of ribosome	*	*	*	*	*	**
PADG_02555	Nucleic acid-binding protein	4268	1.22	***	0.654	-	Nucleotide binding,nucleic acid binding	Yes	Yes	*	*	*	**
PADG_07418	Superoxide dismutase [Cu-Zn]	12,014	1.25	***	***	-	Superoxide dismutase activity,metal ion binding	Yes	*	*	*	*	**
PADG_02652	Grp1p	5794	1.27	***	0.839	-	Nucleotide binding,single-stranded telomeric DNA binding	*	Yes	*	*	*	**
PADG_01407	40S ribosomal protein	9378	1.28	***	***	-	RNA binding,structural constituent of ribosome	*	Yes	*	*	*	**
PADG_06515	Suaprga1 •	22,754	1.32	***	0.754	-	NDA	*	*	*	*	*	**
PADG_02017	Calmodulin	43,048	1.35	***	0.744	-	Calcium ion binding	*	Yes	*	Yes	*	**
PADG_08369	Hsp60-like protein	17,604	1.41	***	***	-	DNA replication origin binding, single-stranded DNA binding, ATP binding, ATPase activity	Yes	Yes	*	*	*	**
PADG_05160	Dipeptidyl peptidase 3	2563	1.44	***	***	-	Dipeptidyl-peptidase activity,metal ion binding	*	Yes	*	Yes	*	**
PADG_07524	Nucleoside diphosphate kinase	37,920	1.47	***	***	-	Nucleoside diphosphate kinase activity,ATP binding	Yes	Yes	*	Yes	*	**
PADG_01605	Polyubiquitin	4911	1.48	***	***	-	NDA	*	Yes	*	*	*	**
PADG_03149	Aminopeptidase	2712	1.49	***	***	-	Metallopeptidase activity, zinc ion binding	*	Yes	*	Yes	Yes	**
PADG_03841	Protein disulfide-isomerase domain	22,371	1.60	0.726	***	-	Protein disulfide isomerase activity	*	Yes	*	*	*	**
PADG_07782	Deoxyuridine 5-triphosphate nucleotidohydrolase	7471	1.61	***	0.601	-	dUTP diphosphatase activity	*	Yes	*	*	*	**
PADG_07420	Transaldolase	7105	1.63	***	***	-	Sedoheptulose-7-phosphate:D-glyceraldehyde-3-phosphate glyceronetransferase activity	*	Yes	*	*	*	**

PADG_04148	Vacuolar alpha mannosidase	5961	1.70	***	***	-	Alpha-mannosidase activity,zinc ion binding,carbohydrate binding	*	Yes	*	*	*	**
PADG_08054	Malate dehydrogenase, NAD-dependent	4526	1.81	***	***	-	L-malate dehydrogenase activity	Yes	*	Yes	Yes	Yes	**
PADG_02785	Heat shock protein Hsp88	5877	1.83	***	***	-	ATP binding	Yes	Yes	*	Yes	*	**
PADG_00688	F-type H+-transporting ATPase subunit H	8080	1.96	***	0.827	-	NDA	*	Yes	*	*	*	**
PADG_05032	Hsp90 binding co-chaperone (Sba1)	8142	2.14	***	***	-	NDA	Yes	*	*	Yes	*	**
PADG_01479	Gamma-glutamyl transpeptidase 2	6243	2.43	***	***	Bailão *et al*., 2015	Gamma-glutamyltransferase activity	Yes	*	*	*	*	**
PADG_00921	Hypothetical protein •	3213	2.51	***	***	-	NDA	*	*	*	*	*	**
PADG_04059	Enolase	9477	2.52	***	***	Donofrio *et al*., 2009; Nogueira *et al*., 2010; Marcos *et al*., 2012	Magnesium ion binding, phosphopyruvate hydratase activity	Yes	Yes	*	Yes	*	**
PADG_01711	Hsp90 co-chaperone AHA1	11,850	2.54	***	***	-	ATPase activator activity	*	Yes	*	Yes	*	**
PADG_01706	Fructose-1,6-bisphosphatase	13,110	2.58	***	***	-	Fructose 1,6-bisphosphate 1-phosphatase activity	*	Yes	*	*	*	**
PADG_04710	2-methylcitrate synthase, mitochondrial	17,077	2.84	***	***	-	Citrate (Si)-synthase activity, 2-methylcitrate synthase activity	Yes	Yes	Yes	Yes	Yes	**
PADG_02343	MYG1 protein •	7766	2.95	***	***	-	NDA	*	*	*	*	*	**
PADG_07422	Serine proteinase	3358	3.06	0.669	***	Parente *et al*., 2010; Pigosso *et al*., 2017	Serine-type endopeptidase activity	*	Yes	*	*	*	**
PADG_06490	Formamidase	12,362	3.15	***	***	Borges *et al*., 2010	Hydrolase activity, acting on carbon-nitrogen (but not peptide) bonds, in linear amides	Yes	Yes	Yes	Yes	Yes	**
PADG_02446	60S acidic ribosomal protein P2	29,077	3.61	***	***	-	Structural constituent of ribosome	*	Yes	*	Yes	*	**
PADG_02735	Proteasome component PRE6	13,436	3.91	***	***	-	Threonine-type endopeptidase activity	*	Yes	*	*	*	**
PADG_04288	Endoribonuclease L-PSP	31,240	3.94	***	0.874	-	NDA	*	Yes	*	Yes	Yes	**

PADG_08118	Hsp72-like protein	49,968	3.97	***	***	-	ATP binding	*	Yes	*	*	Yes	**
PADG_05855	Lactonohydrolase	8467	4.07	***	***	-	NDA	*	*	*	*	*	**
PADG_11950	Transcription initiation factor TFIID/TFIIF subunit •	13,634	4.72	***	***	-	NDA	*	*	*	*	*	**
PADG_05750	Putative cytochrome c oxidase subunit VIa	23,847	5.07	***	0.722	-	Cytochrome-c oxidase activity	Yes	Yes	*	*	*	**
PADG_00663	Homoserine dehydrogenase	7981	5.12	***	0.651	-	Homoserine dehydrogenase activity,NADP binding	*	Yes	*	Yes	*	**
PADG_06494	Dihydrolipoyl dehydrogenase	32,031	5.37	***	***	Landgraf *et al*., 2017	Dihydrolipoyl dehydrogenase activity, glycine dehydrogenase (decarboxylating) activity, oxoglutarate dehydrogenase (succinyl-transferring) activity, pyruvate dehydrogenase activity, flavin adenine dinucleotide binding	Yes	Yes	*	Yes	Yes	**
PADG_05798	Single-strand binding protein family	41,240	7.76	***	0.809	-	Single-stranded DNA binding	*	Yes	*	*	*	**
PADG_03559	Cytochrome b5 #	17,493	30.67	***	0.688	-	Heme binding, metal ion binding	*	Yes	*	*	*	**
PADG_00026	Cytidine deaminase •	4546	BPS	***	***	-	Cytidine deaminase activity, zinc ion binding,	*	*	*	*	*	**
PADG_00060	MGS207 protein •	1122	BPS	***	***	-	NDA	*	*	*	*	*	**
PADG_00210	Glycine dehydrogenase	1077	BPS	***	***	-	Glycine dehydrogenase (decarboxylating) activity	*	Yes	*	*	*	**
PADG_00211	DUF427 domain protein •	3310	BPS	***	***	-	NDA	*	*	*	*	*	**
PADG_00220	CBF/NF-Y family transcription factor •	1323	BPS	***	***	-	Core promoter binding, RNA polymerase II transcription factor activity, TBP-class protein binding, involved in preinitiation complex	*	*	*	*	*	**

							assembly, chromatin binding, transcription coactivator activity, transcription corepressor activity						
PADG_00451	Glucose-6-phosphate isomerase	1011	BPS	***	***	-	glucose-6-phosphate isomerase activity	*	Yes	*	*	*	**
PADG_00514	60S ribosomal protein L16 •	1435	BPS	***	***	-	Structural constituent of ribosome	*	*	*	*	*	**
PADG_00615	Proteasome subunit alpha type-C7 •	1010	BPS	***	***	-	Threonine-type endopeptidase activity	*	*	*	*	*	**
PADG_00676	RNAPII degradation factor def1	1191	BPS	***	***	-	NDA	*	Yes	*	*	*	**
PADG_00809	Ubiquitin-conjugating enzyme •	2155	BPS	***	0.825	-	NDA	*	*	*	*	*	**
PADG_00822	Glutaminase A	786	BPS	0.730	***	-	NDA	*	Yes	*	*	*	**
PADG_00888	Argininosuccinate synthase •	804	BPS	***	***	-	Argininosuccinate synthase activity, ATP binding	*	*	*	*	*	**
PADG_00912	UDP-galactopyranose mutase	1287	BPS	***	***	-	Oxidoreductase activity	*	Yes	*	*	*	**
PADG_00988	Ribonuclease T2 •	923	BPS	***	0.614	-	RNA binding, ribonuclease T2 activity	*	*	*	*	*	**
PADG_01404	Aspartate aminotransferase	740	BPS	***	0.662	-	L-aspartate:2-oxoglutarate aminotransferase activity, pyridoxal phosphate binding, L-phenylalanine:2-oxoglutarate aminotransferase activity	*	Yes	*	*	*	**
PADG_01626	Iron donor protein CyaY •#	5703	BPS	***	***	-	Ferroxidase activity, ferric iron binding	*	*	*	*	*	**
PADG_01644	Vesicle transport v-SNARE protein vti1 •	1227	BPS	***	***	-	NDA	*	*	*	*	*	**
PADG_01654	40S ribosomal protein S6-A •	1333	BPS	***	***	-	Structural constituent of ribosome	*	*	*	*	*	**
PADG_01665	Kynurenine-oxoglutarate transaminase •	1278	BPS	***	***	-	Kynurenine-oxoglutarate transaminase	*	*	*	*	*	**

							activity, pyridoxal phosphate binding, 2-aminoadipate transaminase activity						
PADG_01871	3-oxoacyl-(Acyl-carrier-protein) reductase •	1652	BPS	***	0.895	-	NDA	*	*	*	*	*	**
PADG_01949	Translation elongation factor Tu •	2648	BPS	***	0.751	Marcos *et al*., 2016; Marcos *et al*., 2019	Translation elongation factor activity, GTPase activity, GTP binding	*	*	*	*	*	**
PADG_02260	Succinate-CoA ligase [ADP-forming] subunit alpha, mitochondrial	2156	BPS	***	0.644	-	Ligase activity, cofactor binding	*	Yes	*	*	*	**
PADG_02561	ATP synthase subunit alpha, mitochondrial	2772	BPS	***	***	-	ATP binding, proton-transporting ATP synthase activity, rotational mechanism	*	Yes	*	*	*	**
PADG_02763	Cyclin-dependent kinase regulatory subunit •	3528	BPS	***	0.773	-	Kinase activity, cyclin-dependent protein serine/threonine kinase regulator activity	*	*	*	*	*	**
PADG_02845	Diploid state maintenance protein chpA •	1291	BPS	***	0.829	-	NDA	*	*	*	*	*	**
PADG_02862	Glucan 1,3-beta-glucosidase	1997	BPS	0.871	***	-	1,3-beta-glucanosyltransferase activity, glucan endo-1,3-beta-D-glucosidase activity	Yes	*	*	*	*	**
PADG_03121	Domain-containing protein •	1070	BPS	***	0.811	-	NDA	*	*	*	*	*	**
PADG_03278	Inositol-3-phosphate synthase •	885	BPS	***	0.640	-	Inositol-3-phosphate synthase activity	*	*	*	*	*	**
PADG_03526	M protein repeat protein •	637	BPS	***	***	-	NDA	*	*	*	*	*	**
PADG_03778	60S ribosomal protein L10-A •	1463	BPS	***	0.678	-	Structural constituent of ribosome	*	*	*	*	*	**
PADG_03830	Actin-interacting protein	637	BPS	***	***	-	NDA	Yes	*	*	*	*	**
PADG_03852	LiPid Depleted family member •#	779	BPS	***	0.692	-	Iron ion binding, iron-sulfur cluster binding	*	*	*	*	*	Yes
PADG_03856	60S ribosomal protein L15 •	1833	BPS	***	***	-	Structural constituent of ribosome	*	*	*	*	*	**
PADG_04030	60S acidic ribosomal protein P0	1335	BPS	***	***	-	Structural constituent of ribosome, large ribosomal subunit rRNA binding	*	Yes	*	*	*	**

PADG_04242	Sulfhydryl oxidase •	5011	BPS	***	***	-	Thiol oxidase activity	*	*	*	*	*	**
PADG_04475	Ribosomal protein S36, mitochondrial •	3489	BPS	***	0.791	-	NDA	*	*	*	*	*	**
PADG_04516	NADP-specific glutamate dehydrogenase	1563	BPS	***	***	-	Glutamate dehydrogenase (NAD+) activity	*	*	Yes	*	*	**
PADG_04588	60S ribosomal protein L22	3027	BPS	***	***	-	Structural constituent of ribosome	*	Yes	*	Yes	*	**
PADG_04934	RNP domain protein	919	BPS	***	***	-	Nucleotide binding, nucleic acid binding	*	*	*	Yes	*	**
PADG_05239	Tubulin binding cofactor A •	2900	BPS	***	***	-	NDA	*	*	*	*	*	**
PADG_05277	Serine hydroxymethyltransferase	655	BPS	***	***	-	Glycine hydroxymethyltransferase activity, pyridoxal phosphate binding	Yes	*	*	*	*	**
PADG_05321	Mitochondrial nuclease #	1132	BPS	***	***	-	Single-stranded DNA endodeoxyribonuclease activity, nucleic acid binding, endoribonuclease activity, metal ion binding	*	Yes	*	*	*	**
PADG_05683	Cell division control protein 48 •	1683	BPS	***	***	-	ATP binding, protein phosphatase type 1 regulator activity, ATPase activity	*	*	*	*	*	**
PADG_06155	Palmitoyl-protein thioesterase	1632	BPS	0.808	***	-	Palmitoyl-(protein) hydrolase activity	*	Yes	*	*	*	**
PADG_06273	Calcineurin subunit B •	2812	BPS	***	***	-	Phosphoprotein phosphatase activity, calcium ion binding, calcium-dependent protein serine/threonine phosphatase regulator activity	*	*	*	*	*	**
PADG_06382	3-methyl-2-oxobutanoate hydroxymethyltransferase •	711	BPS	***	0.685	-	Transferase activity, transferring acyl groups other than amino-acyl groups	*	*	*	*	*	**

PADG_06671	3-isopropylmalate dehydrogenase A	1032	BPS	***	***	-	Magnesium ion binding, 3-isopropylmalate dehydrogenase activity, NAD binding	*	Yes	*	*	*	**
PADG_06726	60S ribosomal protein L17 •	1694	BPS	***	0.869	-	Structural constituent of ribosome	*	*	*	*	*	**
PADG_06838	40S ribosomal protein S5	3023	BPS	***	0.632	-	RNA binding, structural constituent of ribosome	*	Yes	*	*	*	**
PADG_06997	Nuclear cap-binding protein •	2485	BPS	***	0.665	-	Nucleotide binding, nucleic acid binding, peptidyl-prolyl cis-trans isomerase activity	*	*	*	*	*	**
PADG_07264	Stress responsive A/B barrel domain protein •	3637	BPS	***	***	-	NDA	*	*	*	*	*	**
PADG_07870	30S ribosomal protein S7 •	1112	BPS	***	***	-	Structural constituent of ribosome	*	*	*	*	*	**
PADG_07888	Eukaryotic translation initiation factor 5A	4225	BPS	***	***	-	Translation initiation factor activity, translation elongation factor activity, ribosome binding	Yes	*	*	*	*	**
PADG_08045	Branched-chain amino acid aminotransferase •	795	BPS	***	0.682	-	Branched-chain-amino-acid transaminase activity	*	*	*	*	*	**
PADG_08098	Adenylate kinase 1 •	1995	BPS	***	0.666	-	Adenylate kinase activity, ATP binding	*	*	*	*	*	**
PADG_08108	Coproporphyrinogen III oxidase •#	1114	BPS	***	***	-	Coproporphyrinogen oxidase activity	*	*	*	*	*	**
PADG_08212	Hypothetical protein •	4444	BPS	***	0.637	-	NDA	*	*	*	*	*	**
PADG_08244	60S acidic ribosomal protein P1	14,128	BPS	***	***	-	Structural constituent of ribosome	*	Yes	*	*	*	**
PADG_08270	UBX domain-containing protein •#	1143	BPS	***	0.891	-	Metal ion binding	*	*	*	*	*	**
PADG_08328	5-methyltetrahydropteroyltriglutamate-homocysteine S-methyltransferase •	756	BPS	***	***	-	5-methyltetrahydropteroyltriglutamate-homocysteine S-methyltransferase	*	*	*	*	*	**

							activity, zinc ion binding,						
PADG_08376	Aspartate-semialdehyde dehydrogenase	2046	BPS	***	0.618	-	N-acetyl-gamma-glutamyl-phosphate reductase activity, aspartate-semialdehyde dehydrogenase activity, NADP binding, NAD binding	*	Yes	*	*	*	**
PADG_08391	Plasma membrane ATPase #	517	BPS	***	0.715	-	ATP binding, hydrogen-exporting ATPase activity, phosphorylative mechanism, metal ion binding	*	Yes	*	*	*	**
PADG_08466	Homogentisate 1,2-dioxygenase #	784	BPS	***	0.601	-	Homogentisate 1,2-dioxygenase activity	*	*	Yes	*	*	Yes
PADG_08468	4-hydroxyphenylpyruvate dioxygenase •#	897	BPS	***	***	-	4-hydroxyphenylpyruvate dioxygenase activity, metal ion binding	*	*	*	*	*	Yes
PADG_08587	FK506-binding protein	3833	BPS	0.869	***	-	NDA	*	Yes	*	*	*	**
PADG_08599	DnaJ domain protein •	1141	BPS	***	***	-	NDA	*	*	*	*	*	**
PADG_11679	Proliferating cell nuclear antigen (pcna)	1358	BPS	***	***	-	DNA binding, DNA polymerase processivity factor activity	*	Yes	*	*	*	**
PADG_11711	ATP-dependent RNA helicase eIF4A •	1151	BPS	***	***	-	Nucleic acid binding, helicase activity, ATP binding	*	*	*	*	*	**
PADG_12077	Actin	1073	BPS	***	***	-	structural constituent of cytoskeleton, ATP binding	*	Yes	*	*	*	**
PADG_12252	Phosphotransferase enzyme family protein •	987	BPS	***	***	-	NDA	*	*	*	*	*	**
PADG_12253	60S ribosomal protein L3	1229	BPS	***	***	-	Structural constituent of ribosome, carbon-sulfur lyase activity	*	Yes	*	*	*	**
PADG_12365	40S ribosomal protein S8-A •	890	BPS	***	0.792	-	Structural constituent of ribosome	*	*	*	*	*	**

The table was organized in ascending order of BPS:Fe ratio's values.

[a]Protein accession number in NCBI, available at https://www.ncbi.nlm.nih.gov/protein.

[b]Description of the protein in the *Paracoccidioides* spp. databank, available on https://www.uniprot.org/proteomes/?query=paracoccidioides&sort=score.

•Points out that the protein was exclusively identified in the present work.

#Points out that the protein potentially binds to iron.

[c]The protein identification's score number is obtained by a multi-step process carried out by PLGS. Only data with ≥ 95% confidence level and a false discovery ratio ≤ 4% were used in the present study.

[d]Ratio between quantification of proteins identified in iron restriction/iron sufficiency conditions. Values ≥ 2.0 indicate upregulated proteins; Values ≤ 0.5 indicate downregulated proteins; BPS indicates that the protein was identified only upon iron restriction condition.

[e]Prediction of signal peptide presence; score must be ≥ 0.45; prediction performed by SignalP 4.1 available at http://www.cbs.dtu.dk/services/SignalP-4.1/; (***) indicates that the signal peptide was not identified.

[f]Prediction of protein secretion by non-classical pathways whose score must be ≥ 0.6; prediction performed by SecretomeP 2.0 available at http://www.cbs.dtu.dk/services/SecretomeP/. (***) indicates that the protein was not predicted as secreted.

[g]Secretion evidence in *Paracoccidioides*'s literature. Searches were performed on the PUBMED database in the period 07/2019 – 07/2021. The terms used in the searches were those in the column "description" + Paracoccidioides. (-) indicates that no evidence for protein secretion in literature was found.

[h]Molecular function described in ParaDB. Available on http://paracoccidioides.com/. NDA indicates that are not descriptions available regarding molecular functions for the protein in this database.

[i]Proteins identified in the work of Weber *et al.*, 2012. Available on https://journals.plos.org/plosone/article?id=10.1371/journal.pone.0052470. (*) indicates that the protein was not identified in the cited study.

[j]Proteins identified in the work of Vallejo *et al*., 2012. Available on https://pubs.acs.org/doi/10.1021/pr200872s. (*) indicates that the protein was not identified in the cited study.

[k]Proteins identified in the work of Chaves *et al*., 2015. Available on https://bmcmicrobiol.biomedcentral.com/articles/10.1186/s12866-015-0393-9. (*) indicates that the protein was not identified in the cited study.

[l]Proteins identified in the work of Oliveira *et al*., 2018. Available on https://www.sciencedirect.com/science/article/pii/S187861461830062X?via%3Dihub. (*) indicates that the protein was not identified in the cited study.

[m]Proteins identified in the work of Moreira *et al*., 2020. Available on https://www.frontiersin.org/articles/10.3389/fmicb.2019.02968/full. (*) indicates that the protein was not identified in the cited study.

[n]Iron-binding's proteins predicted according to Tristão *et al*., 2015. Available on https://doi.org/10.3389/fmicb.2014.00761. (**) indicates that the protein was not predicted as iron bindind.

Supplementary Table 3. Up-regulated proteins identified in exoproteome of *P. brasiliensis* (Pb18) after culturing yeast cells for 48 hours at 37 ºC in MMcM liquid medium with Fe restriction (BPS 50 µM) or Fe supplementation [Fe(NH4)2(SO4)2.6H2O 10 µM]

**Accession[a]**	**Description[b]**	**Score[c]**	**BPS:Fe Ratio[d]**	**SignalP ≥ 0.45[e]**	**SecretomeP ≥ 0.6[f]**	**Evidence of secretion in *Paracoccidioides* spp. literature[g]**	**Weber *et al.*, 2012[h]**	**Vallejo *et al.*, 2012[i]**	**Chaves *et al.*, 2015[j]**	**Oliveira *et al.*, 2018[k]**	**Moreira *et al.*, 2020[l]**	**Tristão *et al.*, 2015[m]**
PADG_05032	Hsp90 binding co-chaperone (Sba1)	8142	2.14	***	***	-	Yes	*	*	Yes	*	**
PADG_01479	Gamma-glutamyl transpeptidase 2	6243	2.43	***	***	Bailão *et al.*, 2015	Yes	*	*	*	*	**
PADG_00921	Hypothetical protein •	3213	2.51	***	***	-	*	*	*	*	*	**
PADG_04059	Enolase	9477	2.52	***	***	Donofrio *et al.*, 2009; Nogueira *et al.*, 2010; Marcos *et al.*, 2012	Yes	Yes	*	Yes	*	**
PADG_01711	Hsp90 co-chaperone AHA1	11850	2.54	***	***	-	*	Yes	*	Yes	*	**
PADG_01706	Fructose-1,6-bisphosphatase	13110	2.58	***	***	-	*	Yes	*	*	*	**
PADG_04710	2-methylcitrate synthase, mitochondrial	17077	2.84	***	***	-	Yes	Yes	Yes	Yes	Yes	**
PADG_02343	MYG1 protein •	7766	2.95	***	***	-	*	*	*	*	*	**
PADG_07422	Serine proteinase	3358	3.06	0.669	***	Parente *et al.*, 2010; Pigosso *et al.*, 2017	*	Yes	*	*	*	**
PADG_06490	Formamidase	12362	3.15	***	***	Borges *et al.*, 2010	Yes	Yes	Yes	Yes	Yes	**
PADG_02446	60S acidic ribosomal protein P2	29077	3.61	***	***	-	*	Yes	*	Yes	*	**
PADG_02735	Proteasome component PRE6	13436	3.91	***	***	-	*	Yes	*	*	*	**
PADG_04288	Endoribonuclease L-PSP	31240	3.94	***	0.874	-	*	Yes	*	Yes	Yes	**
PADG_08118	Hsp72-like protein	49968	3.97	***	***	-	*	Yes	*	*	Yes	**
PADG_05855	Lactonohydrolase	8467	4.07	***	***	-	*	*	*	*	*	**
PADG_11950	Transcription initiation factor TFIID/TFIIF subunit •	13634	4.72	***	***	-	*	*	*	*	*	**
PADG_05750	Putative cytochrome c oxidase subunit VIa	23847	5.07	***	0.722	-	Yes	Yes	*	*	*	**

PADG_00663	Homoserine dehydrogenase	7981	5.12	***	0.651	-	*	Yes	*	Yes	*	**
PADG_06494	Dihydrolipoyl dehydrogenase	32031	5.37	***	***	Landgraf *et al.*, 2017	Yes	Yes	*	Yes	Yes	**
PADG_05798	Single-strand binding protein family	41240	7.76	***	0.809	-	*	Yes	*	*	*	**
PADG_03559	Cytochrome b5 #	17493	30.67	***	0.688	-	*	Yes	*	*	*	**
PADG_00026	Cytidine deaminase •	4546	BPS	***	***	-	*	*	*	*	*	**
PADG_00060	MGS207 protein •	1122	BPS	***	***	-	*	*	*	*	*	**
PADG_00210	Glycine dehydrogenase	1077	BPS	***	***	-	*	Yes	*	*	*	**
PADG_00211	DUF427 domain protein •	3310	BPS	***	***	-	*	*	*	*	*	**
PADG_00220	CBF/NF-Y family transcription factor •	1323	BPS	***	***	-	*	*	*	*	*	**
PADG_00451	Glucose-6-phosphate isomerase	1011	BPS	***	***	-	*	Yes	*	*	*	**
PADG_00514	60S ribosomal protein L16 •	1435	BPS	***	***	-	*	*	*	*	*	**
PADG_00615	Proteasome subunit alpha type-C7 •	1010	BPS	***	***	-	*	*	*	*	*	**
PADG_00676	RNAPII degradation factor def1	1191	BPS	***	***	-	*	Yes	*	*	*	**
PADG_00809	Ubiquitin-conjugating enzyme •	2155	BPS	***	0.825	-	*	*	*	*	*	**
PADG_00822	Glutaminase A	786	BPS	0.730	***	-	*	Yes	*	*	*	**
PADG_00888	Argininosuccinate synthase •	804	BPS	***	***	-	*	*	*	*	*	**
PADG_00912	UDP-galactopyranose mutase	1287	BPS	***	***	-	*	Yes	*	*	*	**
PADG_00988	Ribonuclease T2 •	923	BPS	***	0.614	-	*	*	*	*	*	**
PADG_01404	Aspartate aminotransferase	740	BPS	***	0.662	-	*	Yes	*	*	*	**
PADG_01626	Iron donor protein CyaY •#	5703	BPS	***	***	-	*	*	*	*	*	**
PADG_01644	Vesicle transport v-SNARE protein vti1 •	1227	BPS	***	***	-	*	*	*	*	*	**
PADG_01654	40S ribosomal protein S6-A •	1333	BPS	***	***	-	*	*	*	*	*	**
PADG_01665	Kynurenine-oxoglutarate transaminase •	1278	BPS	***	***	-	*	*	*	*	*	**
PADG_01871	3-oxoacyl-(Acyl-carrier-protein) reductase •	1652	BPS	***	0.895	-	*	*	*	*	*	**
PADG_01949	Translation elongation factor Tu •	2648	BPS	***	0.751	Marcos *et al.*, 2016; Marcos *et al.*, 2019	*	*	*	*	*	**
PADG_02260	Succinate-CoA ligase [ADP-forming] subunit alpha, mitochondrial	2156	BPS	***	0.644	-	*	Yes	*	*	*	**
PADG_02561	ATP synthase subunit alpha, mitochondrial	2772	BPS	***	***	-	*	Yes	*	*	*	**
PADG_02763	Cyclin-dependent kinase regulatory subunit •	3528	BPS	***	0.773	-	*	*	*	*	*	**
PADG_02845	Diploid state maintenance protein chpA •	1291	BPS	***	0.829	-	*	*	*	*	*	**
PADG_02862	Glucan 1,3-beta-glucosidase	1997	BPS	0.871	***	-	Yes	*	*	*	*	**
PADG_03121	Domain-containing protein •	1070	BPS	***	0.811	-	*	*	*	*	*	**
PADG_03278	Inositol-3-phosphate synthase •	885	BPS	***	0.640	-	*	*	*	*	*	**
PADG_03526	M protein repeat protein •	637	BPS	***	***	-	*	*	*	*	*	**
PADG_03778	60S ribosomal protein L10-A •	1463	BPS	***	0.678	-	*	*	*	*	*	**

PADG_03830	Actin-interacting protein	637	BPS	***	***	-	Yes	*	*	*	*	**
PADG_03852	LiPid Depleted family member •#	779	BPS	***	0.692	-	*	*	*	*	*	**Yes**
PADG_03856	60S ribosomal protein L15 •	1833	BPS	***	***	-	*	*	*	*	*	**
PADG_04030	60S acidic ribosomal protein P0	1335	BPS	***	***	-	*	Yes	*	*	*	**
PADG_04242	Sulfhydryl oxidase •	5011	BPS	***	***	-	*	*	*	*	*	**
PADG_04475	Ribosomal protein S36, mitochondrial •	3489	BPS	***	0.791	-	*	*	*	*	*	**
PADG_04516	NADP-specific glutamate dehydrogenase	1563	BPS	***	***	-	*	*	Yes	*	*	**
PADG_04588	60S ribosomal protein L22	3027	BPS	***	***	-	*	Yes	*	Yes	*	**
PADG_04934	RNP domain protein	919	BPS	***	***	-	*	*	*	Yes	*	**
PADG_05239	Tubulin binding cofactor A •	2900	BPS	***	***	-	*	*	*	*	*	**
PADG_05277	Serine hydroxymethyltransferase	655	BPS	***	***	-	Yes	*	*	*	*	**
PADG_05321	Mitochondrial nuclease #	1132	BPS	***	***	-	*	Yes	*	*	*	**
PADG_05683	Cell division control protein 48 •	1683	BPS	***	***	-	*	*	*	*	*	**
PADG_06155	Palmitoyl-protein thioesterase	1632	BPS	0.808	***	-	*	Yes	*	*	*	**
PADG_06273	Calcineurin subunit B •	2812	BPS	***	***	-	*	*	*	*	*	**
PADG_06382	3-methyl-2-oxobutanoate hydroxymethyltransferase •	711	BPS	***	0.685	-	*	*	*	*	*	**
PADG_06671	3-isopropylmalate dehydrogenase A	1032	BPS	***	***	-	*	Yes	*	*	*	**
PADG_06726	60S ribosomal protein L17 •	1694	BPS	***	0.869	-	*	*	*	*	*	**
PADG_06838	40S ribosomal protein S5	3023	BPS	***	0.632	-	*	Yes	*	*	*	**
PADG_06997	Nuclear cap-binding protein •	2485	BPS	***	0.665	-	*	*	*	*	*	**
PADG_07264	Stress responsive A/B barrel domain protein •	3637	BPS	***	***	-	*	*	*	*	*	**
PADG_07870	30S ribosomal protein S7 •	1112	BPS	***	***	-	*	*	*	*	*	**
PADG_07888	Eukaryotic translation initiation factor 5A	4225	BPS	***	***	-	Yes	*	*	*	*	**
PADG_08045	Branched-chain amino acid aminotransferase •	795	BPS	***	0.682	-	*	*	*	*	*	**
PADG_08098	Adenylate kinase 1 •	1995	BPS	***	0.666	-	*	*	*	*	*	**
PADG_08108	Coproporphyrinogen III oxidase •#	1114	BPS	***	***	-	*	*	*	*	*	**
PADG_08212	Hypothetical protein •	4444	BPS	***	0.637	-	*	*	*	*	*	**
PADG_08244	60S acidic ribosomal protein P1	14128	BPS	***	***	-	*	Yes	*	*	*	**
PADG_08270	UBX domain-containing protein •#	1143	BPS	***	0.891	-	*	*	*	*	*	**
PADG_08328	5-methyltetrahydropteroyltriglutamate-homocysteine S-methyltransferase •	756	BPS	***	***	-	*	*	*	*	*	**
PADG_08376	Aspartate-semialdehyde dehydrogenase	2046	BPS	***	0.618	-	*	Yes	*	*	*	**

PADG_08391	Plasma membrane ATPase #	517	BPS	***	0.715	-	*	Yes	*	*	*	**
PADG_08466	Homogentisate 1,2-dioxygenase #	784	BPS	***	0.601	-	*	*	Yes	*	*	**Yes**
PADG_08468	4-hydroxyphenylpyruvate dioxygenase •#	897	BPS	***	***	-	*	*	*	*	*	**Yes**
PADG_08587	FK506-binding protein	3833	BPS	0.869	***	-	*	Yes	*	*	*	**
PADG_08599	DnaJ domain protein •	1141	BPS	***	***	-	*	*	*	*	*	**
PADG_11679	Proliferating cell nuclear antigen (pcna)	1358	BPS	***	***	-	*	Yes	*	*	*	**
PADG_11711	ATP-dependent RNA helicase eIF4A •	1151	BPS	***	***	-	*	*	*	*	*	**
PADG_12077	Actin	1073	BPS	***	***	-	*	Yes	*	*	*	**
PADG_12252	Phosphotransferase enzyme family protein •	987	BPS	***	***	-	*	*	*	*	*	**
PADG_12253	60S ribosomal protein L3	1229	BPS	***	***	-	*	Yes	*	*	*	**
PADG_12365	40S ribosomal protein S8-A •	890	BPS	***	0.792	-	*	*	*	*	*	**

[a]Protein accession number in NCBI, available at https://www.ncbi.nlm.nih.gov/protein.

[b]Description of the protein in the *Paracoccidioides* spp. databank, available in https://www.uniprot.org/proteomes/?query=paracoccidioides&sort=score.

•Points out that the protein was exclusively identified in the present work.

#Points out that the protein potentially binds to iron.

[c]The protein identification's score number is obtained by a multi-step process carried out by PLGS. Only data with ≥ 95% confidence level and a false discovery ratio ≤ 4% were used in the present study.

[d]Ratio between quantification of proteins identified in iron restriction/iron sufficiency conditions. Values ≥ 2.0 indicate upregulated proteins; BPS indicates that the protein was identified only upon iron restriction condition.

[e]Prediction of signal peptide presence; score must be ≥ 0.45; prediction performed by SignalP 4.1 available at http://www.cbs.dtu.dk/services/SignalP-4.1/; (***) indicates that the signal peptide was not identified.

[f]Prediction of protein secretion by non-classical pathways whose score must be ≥ 0.6; prediction performed by SecretomeP 2.0 available at http://www.cbs.dtu.dk/services/SecretomeP/. (***) indicates that the protein was not predicted as secreted.

[g]Secretion evidence in *Paracoccidioides*'s literature. Searches were performed on the PUBMED database in the period 07/2019 – 07/2021. The terms used in the searches were those in the column "description" + Paracoccidioides. (-) indicates that no evidence for protein secretion in literature was found.

[h]Proteins identified in the work of Weber *et al*., 2012. Available in https://journals.plos.org/plosone/article?id=10.1371/journal.pone.0052470. (*) indicates that the protein was not identified in the cited study.

[i]Proteins identified in the work of Vallejo *et al*., 2012. Available in https://pubs.acs.org/doi/10.1021/pr200872s. (*) indicates that the protein was not identified in the cited study.

[j]Proteins identified in the work of Chaves *et al*., 2015. Available in https://bmcmicrobiol.biomedcentral.com/articles/10.1186/s12866-015-0393-9. (*) indicates that the protein was not identified in the cited study.

[k]Proteins identified in the work of Oliveira *et al*., 2018. Available in https://www.sciencedirect.com/science/article/pii/S187861461830062X?via%3Dihub. (*) indicates that the protein was not identified in the cited study.

[l]Proteins identified in the work of Moreira *et al*., 2020. Available in https://www.frontiersin.org/articles/10.3389/fmicb.2019.02968/full. (*) indicates that the protein was not identified in the cited study.

[m]Iron-binding's proteins predicted according to Tristão *et al*., 2015. Available in https://doi.org/10.3389/fmicb.2014.00761. (**) indicates that the protein was not predicted as iron binding.
